# Mitigating the burden of dental caries among adolescents and young adults by targeting social media influencers

**DOI:** 10.3389/froh.2026.1879149

**Published:** 2026-07-24

**Authors:** Saurabh RamBihariLal Shrivastava, Prateek Sudhakar Bobhate, Harshal Gajanan Mendhe, Prithvi Brahmanand Petkar

**Affiliations:** 1 Department of Education Research, School of Higher Education and Research, Datta Meghe Institute of Higher Education and Research, Sawangi, Wardha, Maharashtra, India; 2 Department of Community Medicine, Datta Meghe Medical College, Datta Meghe Institute of Higher Education and Research, Wanadongri, Nagpur, Maharashtra, India; 3 Department of Community Medicine, All India Institute of Medical Sciences, Vijaypur, India

**Keywords:** adolescents, dental caries, influencers, oral health, social media, sugary food, youth

## Abstract

Oral disease among young people is a major global public health concern owing to its magnitude and impact on the health, quality of life, and wellbeing of affected individuals. Modern food marketing approaches have undergone a paradigm shift from traditional advertising toward personalized digital campaigns based on the online experiences of the youth. Marketing by social media influencers is powerful as adolescents consider them relatable and trustworthy peers and not just advertisers. Social media influencers tend to shape identity formation in young people by presenting them with an idealized lifestyle and food consumption norms that they often seek to imitate. Acknowledging the high burden of dental diseases among adolescents and the youth and the growing evidence linking exposure to social media influencer marketing with consumption of unhealthy food and sugary beverages by these users, there is an immense need to take steps to mitigate the magnitude of this problem in the digital era. In conclusion, growing evidence suggests that social media influencers can shape the dietary behaviors of young people and adolescents by promoting unhealthy food and beverage choices. While direct evidence linking influencer exposure to the development of dental caries is scarce, the behavioral pathway of increased consumption of cariogenic foods and beverages represents a significant public health concern. Safeguarding the youth and adolescents from a digitally driven cariogenic environment through multisectoral interventions targeting behavioral risk factors for oral diseases is vital for reducing the future burden of oral disease. Finally, longitudinal studies are warranted to better establish the causal relationships between influencer-driven food marketing, dietary behaviors, and dental caries.

## 1 Introduction

Oral disease among young people is a major global public health concern owing to its magnitude and impact on the health, quality of life, and wellbeing of affected individuals (1–4). The results from a secondary analysis revealed that oral disease continues to affect millions of adolescents and young adults aged 10–24 years regardless of gender, socioeconomic class, and geographical area (1). A meta-analysis conducted among European adolescents reported a high prevalence of dental caries at both the enamel and dentine levels, suggesting that untreated caries is also common in developed nations (2). Another study that analyzed the worldwide dental caries trend highlighted a substantial burden and reported significant inequalities between high-income and low-income population groups (3). An umbrella review stated that poor oral health significantly affects the oral health-related quality of life of adolescents, impacting their social interaction, emotional wellbeing, self-esteem, and daily activities (4).

Although the available evidence in the literature suggests that marketing by social media influencers is linked to unhealthy dietary behaviors among adolescents and youth, these studies are largely observational. In other words, direct evidence demonstrating this association (viz., influencer-generated content independently increases the incidence or progression of dental caries) is limited. Thus, there is a need to conduct longitudinal and interventional studies to strengthen the evidence base. To be precise, the proposed relationship is more indirect, with influencer marketing playing a key role in increasing the consumption of sugary foods and beverages, which is itself a well-established behavioral risk factor for dental caries. Accordingly, this perspective article explores the potential public health implications of this behavioral pathway using the currently available evidence.

## 2 Digital food environment and youth exposure

Modern food marketing approaches have undergone a paradigm shift from traditional advertising toward personalized digital campaigns based on the online experiences of young people (5–7). A policy paper highlighted that digital food marketing is widespread, believable, and difficult for young users to ignore, as advertisements are integrated into entertainment and educational content (5). A meta-analysis reported that food and beverage marketing on digital media significantly influences the food preferences, purchasing requests, and consumption behaviors of children and adolescents (6). Another research study from Mexico argued that digital food marketing is a major public health challenge as companies tend to employ interactive online strategies and adolescent-targeted promotional techniques to market unhealthy food products (7).

Youth and adolescents tend to spend a significant amount of their time on social media platforms, which acts as an opportunity for them to be exposed to food and beverage marketing on these digital platforms (8–10). A study conducted in Australia reported that digital media, i.e., social networking applications and online entertainment platforms, have been flooded with food advertisements targeting children and adolescents (8). A review article found that adolescents are consistently exposed to unhealthy food products through television, online games, YouTube, Instagram, influencer-based social media content, etc. (9). A pilot study conducted among adolescent boys and girls aged 12–16 years found that 67% of the girls viewed products that had excessive total fat, while 42% of the boys were influenced by social media influencers (10).

## 3 Food and beverage promotion by social media influencers

Marketing by social media influencers is an influential aspect of the contemporary digital food environment, with observational studies suggesting that adolescents often perceive influencers to be relatable and trustworthy peers and not just advertisers (11). A cross-sectional study conducted among adolescents revealed that those who were frequently exposed to food and drink marketing on social media had statistically significantly higher consumption of unhealthy foods and sugar-sweetened beverages. This was predominantly because the adolescents admired the influencers and identified with them socially (12). A cross-sectional study that analyzed the content of food and beverage posts by celebrities on Instagram revealed that the majority were of unhealthy products high in sugar, salt, and fat, with confectionery and sugary beverages the most commonly advertised items (13). Another study that focused on posts on TikTok, YouTube, and Instagram by social media influencers in Germany revealed that chocolate, sugar confectionery, beverages, and salty snacks were presented positively and attractively (14).

In a study conducted in Canada, influencer-generated food marketing viewed by children and adolescents primarily included unhealthy foods and beverages, comprising fast food, sugary drinks, and processed snacks (15). The available literature suggests that social media influencers may encourage preferences for sugary foods by making these products appear desirable, entertaining, and socially rewarding (16–18). A qualitative study that included interviews of 17 students in Saudi Arabia stated that influencers often integrate food products into lifestyle-oriented content, such as daily functioning, travel blogs, gaming streams, and fitness reels, which makes the advertisements appear natural rather than commercial (16). A systematic review highlighted the role of giveaways, hashtag challenges, discount coupons, and interactive campaigns on various social media sites in increasing adolescent engagement with unhealthy food brands (17). Another systematic review reported that emotionally appealing storytelling and/or portraying an aspirational lifestyle are commonly used by social media influencers to establish an emotional connection with adolescents (18).

Moreover, food and beverage industries are increasingly collaborating with social media influencers to promote the sale of unhealthy products among youth consumers via targeted and algorithmically driven advertising strategies (19–22). A rapid review of seven studies conducted between 2010 and 2020 found that these companies have expanded their marketing presence and shifted to digital and social media environments (19). The findings of a randomized controlled trial highlighted how food and beverage brands have strategically liaised with social media influencers who possess a strong adolescent following to improve the visibility of and loyalty to their products among youth (20). Another study demonstrated that digital marketing algorithms intensify the exposure of adolescents to unhealthy food advertisements by repeatedly recommending sugary food products. These are usually derived from their engagement patterns and browsing history (21). Finally, owing to weak regulatory frameworks and insufficient monitoring, this influencer-driven advertising allows food companies to market unhealthy products to adolescents and youths (22).

## 4 The psychosocial dimension of influencer-driven consumption

Social media influencers tend to shape identity formation in young people by presenting them with an idealized lifestyle and food consumption norms that they often seek to imitate (23, 24). A cross-sectional survey conducted among 1,085 adolescents from Romania and Serbia indicated that Instagram-based social comparison significantly impacted identity processes. This impact was greater among those who were more susceptible to peer validation (23). Similarly, a scoping review reported that peer influence via social media plays a vital role in determining eating behaviors among adolescents, while digital peer interactions expedite unhealthy food choices and influencer-promoted dietary trends (24).

Moreover, we cannot ignore the psychosocial mechanism of fear of missing out, which is quite common among adolescents in consumption behaviors originating from social media (25). A British longitudinal study reported that social media engagement is linked to poor emotional and behavioral outcomes among adolescents, particularly due to compulsive online comparison and fear of exclusion (25). It is important to acknowledge that influencer-driven food marketing activates reward-related psychological pathways that reinforce unhealthy eating behaviors among adolescents (26). This was evident in a multimethod study conducted among 35 adolescents in Australia, wherein it was revealed that unhealthy food advertisements resulted in high levels of engagement, positive emotional responses, and a strong desire to consume the marketed products (26).

## 5 Preventive and mitigation strategies

Acknowledging the high burden of oral disease among adolescents and the youth and the growing evidence linking exposure to social media influencer marketing with the consumption of unhealthy food and sugary beverages by these users, there is an immense need to take steps to mitigate the magnitude of this problem in the digital era (12, 24, 27–40). Primary interventions should focus on improving the digital health literacy of adolescents. These can start in school settings, wherein adolescents are made aware of the food marketing strategies employed by social media influencers with the intention to minimize their susceptibility to unhealthy food promotions (27). These school-based nutrition literacy initiatives should target reducing sugar intake and oral health awareness, which are expected to reduce cariogenic dietary behaviors (27). Moreover, adolescents should be made aware of hidden advertisements, disclosures about sponsorship, and algorithmically driven promotions. This would play a vital role in improving informed decision-making regarding sugary food consumption (28).

Further, digital education interventions that target parents and families can improve supervision of the online food promotions that adolescents are exposed to, thereby minimizing unhealthy dietary behaviors linked to the use of social media (29). Interventions should also focus on the regulation of influencers and digital food marketing. In a cross-sectional survey conducted in Australia, a strong emphasis was placed on stronger governmental regulations to limit unhealthy food marketing via social media and influencers (30). There is a need to ensure that paid partnerships and sponsored food content are disclosed mandatorily, so that adolescents can understand the commercial intent of advertisements on social media platforms (30). In addition, policies restricting the marketing of unhealthy foods during peak adolescent online engagement times can be effective in reducing repeated exposure to sugary food advertisements (31). There is an immense need to regulate cross-border digital marketing of food, as social media advertisements often bypass national public health policies (32).

Dental and healthcare professionals also have a huge role to play. They should regularly assess the dietary behaviors of adolescents, including the influence of social media, during dental consultations, as envisaged in a survey-based study conducted among 4,414 participants (33). A review article advocated the need for preventive counseling on sugar-containing beverages, as increased consumption has been significantly linked to the development of dental caries and enamel erosion (34). There is a definite need to organize community-based oral health promotion programs targeting adolescents, as these can improve oral health knowledge, behaviors, and preventive practices linked to dental caries (35). Moreover, interdisciplinary collaborations between dentists, nutritionists, psychologists, and educators can strengthen the implementation of preventive strategies, especially those linked to social media-driven dietary habits (36).

Apart from discouraging negative practices and behaviors, it is equally important to promote positive messaging and social media influencers advocating healthy behaviors (12, 24, 37). For instance, cooperative campaigns with health influencers can encourage the consumption of healthy snacks, reduce sugar intake, and promote healthy oral hygiene behaviors, which will counter the digital marketing of unhealthy food (12). These social media campaigns could be led by healthcare professionals and peer role models, as they can positively shape dietary attitudes and preventive oral health practices in youth and adolescents (24). These positive influencers could also encourage water consumption instead of sugar-sweetened beverages, which would significantly reduce cariogenic dietary practices among young people (12). In a study conducted in France, 69 videos on oral health education were identified on TikTok. This showed that social media can also be used for promoting oral health education and preventive messaging among adolescents and young adults (37).

Finally, interventions focused on modifying the behavior of youth and adolescents, such as reducing the frequency of sugary snack exposure instead of focusing on total sugar quantity, should be implemented. This would significantly reduce the probability of dental caries (38). A longitudinal study from China concluded that adolescents who consumed sugary snacks once or more on a daily basis had a significantly higher risk of developing dental caries (38). The creation of a healthier school food environment, through ensuring water availability and increasing water consumption in schools during lunchtime, reduced habitual sugary food consumption among adolescents in California (39).

The basic behavioral approach of reducing screen exposure can significantly decrease exposure to digital food marketing and thereby reduce unhealthy snacking behaviors among children and adolescents, as demonstrated in a randomized, crossover clinical trial (40). Even though the majority of these proposed interventions are supported by public health principles and evidence on unhealthy food marketing, their effectiveness in specifically reducing influencer-mediated dietary behaviors and subsequent dental caries has not yet been comprehensively evaluated. Thus, longitudinal studies are warranted to better establish the causal relationship between influencer-driven food marketing, dietary behavior, and dental caries.

## 6 Conclusion

In conclusion, growing evidence suggests that social media influencers can shape the dietary behavior of youth and adolescents by promoting unhealthy food and beverage choices. While direct evidence linking influencer exposure to the development of dental caries is scarce, the behavioral pathway of increased consumption of cariogenic foods and beverages represents a significant public health concern. Safeguarding youth and adolescents from a digitally driven cariogenic environment through multisectoral interventions targeting behavioral risk factors for oral diseases is vital for reducing the future burden of oral disease.

## Data Availability

The original contributions presented in the study are included in the article/Supplementary Material, further inquiries can be directed to the corresponding author.
